# Feasibility of Conduction System Pacing in Patients with Baseline Bundle Branch Block—A Single-Center Mid-Term Follow-Up Study

**DOI:** 10.3390/jcm13020454

**Published:** 2024-01-14

**Authors:** Catalin Pestrea, Marcela Rusu, Roxana Enache, Ecaterina Cicala, Radu Gavrilescu, Adrian Vaduva, Florin Ortan, Corneliu Iorgulescu, Radu Vatasescu

**Affiliations:** 1 Interventional Cardiology Unit, Brasov County Clinical Emergency Hospital, 500326 Brasov, Romania; marcela.murafa@hotmail.com (M.R.); spac.roxana@yahoo.com (R.E.); cicalaecaterina@gmail.com (E.C.); radu.rgavrilescu@gmail.com (R.G.); tadi_vaduva@yahoo.co.uk (A.V.); ortan.florin@gmail.com (F.O.); 2 Department of Medical and Surgical Specialties, Faculty of Medicine, “Transilvania” University of Brasov, 500019 Brasov, Romania; 3 Electrophysiology and Cardiac Pacing Laboratory, Clinical Emergency Hospital, 014461 Bucharest, Romania; iorgulescu_corneliu@yahoo.com (C.I.); radu_vatasescu@yahoo.com (R.V.); 4 Faculty of Medicine, Carol Davila University of Medicine and Pharmacy, 050474 Bucharest, Romania

**Keywords:** conduction system pacing, bundle branch block, feasibility study, mid-term follow-up

## Abstract

Background: The primary prerequisite for a successful conduction system pacing (CSP) procedure is the integrity of the conduction system, which may be impaired if a baseline bundle branch block (BBB) is present. This study aimed to evaluate the feasibility and mid-term performance of permanent CSP in patients with baseline BBB and to compare the results between left bundle branch block (LBBB) and right bundle branch block (RBBB) patterns. Material and methods: A total of 101 patients with typical BBB and an attempt at CSP were retrospectively reviewed. Procedural characteristics, pacing, sensing parameters, and complications at baseline and after a mid-term follow-up were analyzed. Results: The global procedural success for CSP was 93%. His bundle pacing (HBP) had a significantly lower success rate than left bundle branch area pacing (LBBAP) (50.5% vs. 86%). The paced QRS duration was significantly narrower with HBP. The pacing and sensing thresholds were significantly better with LBBAP. Procedural complications occurred only in the LBBAP group (two acute perforations in the LV cavity and one acute chest pain during lead fixation) without long-term sequelae. The HBP and the LBBAP procedural success rates were higher in the RBBB versus the LBBB group (62.5% vs. 44.9% and 100% vs. 81.5%, respectively). Baseline QRS duration, atrial volumes, and right ventricular diameters were significantly associated with HBP procedural failure. The follow-up pacing and sensing thresholds were similar to the baseline values for all pacing methods and BBB morphology. Only one device-related complication leading to pacing interruption was recorded. Conclusion: In patients with bundle branch blocks, CSP is a feasible procedure associated with a high success rate, stable pacing and sensing parameters, and low complication rates over a mid-term follow-up.

## 1. Introduction

Cardiac pacing has been the only treatment option for patients with severe bradyarrhythmias for over fifty years, significantly impacting mortality and morbidity. The most common site for pacing is in the right ventricle (RV) due to excellent pacing thresholds and lead stability. Unfortunately, increasing scientific data have shown that conventional RV pacing induces significant electrical and mechanical dyssynchrony, leading to a decline in left ventricular (LV) function in up to 20% of the patients, a condition termed pacing-induced cardiomyopathy [1]. The presence of a baseline bundle branch block (BBB) morphology is not only a marker of a diseased conduction system but also has proven to be a predictor of pacing-induced cardiomyopathy occurrence [2]. On the other hand, biventricular pacing has been the gold standard of treatment for patients with BBB, especially LBBB, and decreased ejection fraction, albeit dependent on the patient’s coronary sinus anatomy and having a non-responder rate of up to 30% [3]. Implementing conduction system pacing (CSP) in clinical practice in recent years has improved the outcomes of these patients. Several studies have shown a significant benefit with CSP over RV pacing regarding ejection fraction preservation and, thus, a reduction in pacing-induced cardiomyopathy incidence [4]. Also, several multi-center studies showed that CSP was associated with better LV function outcomes than biventricular pacing in patients with a typical indication for cardiac electrical resynchronization therapy [5].

Currently, two structures of the conduction system are targeted for physiological pacing: the His bundle and the ramifications of the left bundle branch. The prerequisite for a successful procedure is the integrity of the conduction system beyond the pacing site, which may be impaired if a baseline BBB is present. Although a broader native QRS complex has been associated with a lower success rate for both His bundle (HBP) and left bundle branch area pacing (LBBAP), most studies that included these patients have shown good feasibility [6,7]. Nevertheless, there is a concern for the progression of conduction disease beyond the pacing site, which may lead to progressive threshold rise or even the loss of capture over time. Comparison follow-up data between patients with left bundle branch block (LBBB) and right bundle branch block (RBBB) subjected to CSP have been less reported.

This study aimed to evaluate the feasibility and mid-term performance of permanent CSP (HBP and LBBAP) in patients with baseline BBB and to compare the results between LBBB versus RBBB.

## 2. Materials and Methods

### 2.1. Study Design and Patient Population

**Figure 1:**
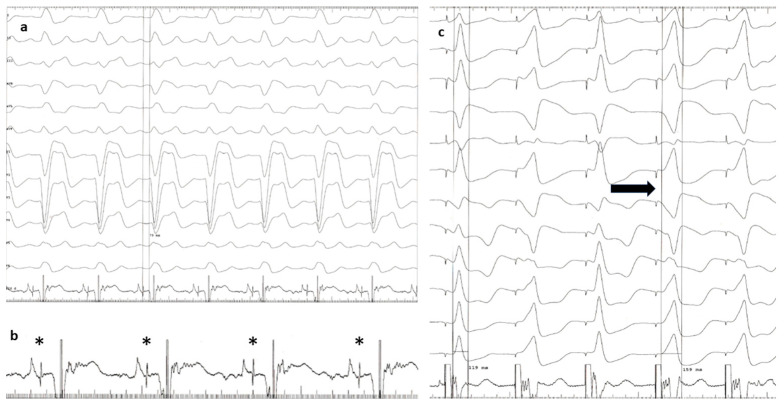
(**a**) A 12-lead ECG (49 mm/s) depicting sinus rhythm with typical LBBB morphology. (**b**) Enhanced intracardiac electrogram showing a sharp His bundle signal (asterisk). (**c**) Decremental pacing with transition from non-selective HBP with LBBB correction to myocardial pacing (black arrow). ECG—electrocardiogram; LBBB—left bundle branch block; HBP—His bundle pacing.

**Figure 2:**
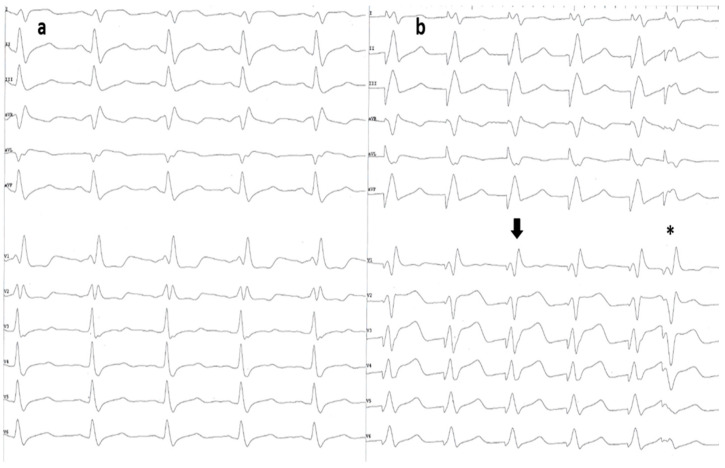
(**a**) A 12-lead ECG depicting sinus rhythm with typical RBBB morphology. (**b**) After the lead was placed in the left bundle branch area, differential pacing was performed to evaluate conduction system capture. After an initial drive train of paced QRS complexes with an RBBB morphology (black arrow), the premature extra stimulus has a different morphology, proving loss of capture of the conduction system and pure myocardial pacing (asterisk). ECG—electrocardiogram; RBBB—right bundle branch block.

This was an analytical, retrospective study with data from a single center. Between July 2018 and December 2021, all patients with baseline BBB who underwent an attempt at CSP for bradyarrhythmias or cardiac resynchronization therapy indications in the Cardiac Pacing Laboratory of the Brașov County Clinical Emergency Hospital in Romania were analyzed for eligibility. Only patients with available 12-lead electrocardiograms and procedural intracardiac electrograms as proof of procedural outcomes were included. Patients without an identified typical BBB pattern on the baseline QRS were excluded. We defined LBBB according to the Strauss criteria, with a QRS duration greater than 140 ms in men or 130 ms in women, QS or rS in leads V1 and V2, and mid-QRS notching or slurring in 2 of leads V1, V2, V5, V6, I, and aVL (Figure 1a) [8]. For RBBB, we used the recommendations for the standardization and interpretation of the electrocardiogram, that is, a QRS duration greater than or equal to 120 milliseconds, an RSR’ pattern in leads V1 and V2, and an S wave of greater duration than the R wave, or an S wave greater than 40 ms in leads 1 and V6 (Figure 2a) [9].

In the end, 101 patients were reviewed in the study. The demographic and clinical characteristics of the patients were recorded at baseline.

### 2.2. Pacing Procedure

The protocol used for CSP at our institution was based on a sequential approach, with HBP attempted first in all patients, followed by LBBAP in case of HBP failure. Briefly, a Medtronic C315 His catheter or a deflectable Medtronic C304 catheter (Medtronic, Minneapolis, MN, USA) with a Select Secure 3830 lead (Medtronic, Minneapolis, MN, USA) inside were advanced at the level of the anteroseptal tricuspid annulus. The His bundle (HB) electrogram was mapped (Figure 1b), and pacing at different amplitudes was performed to evaluate HB capture. If the HB electrogram could not be identified after a maximum of five minutes of fluoroscopy or the HB capture threshold was higher than 2 V at 1 ms pulse duration, the HBP procedure was abandoned, and a delivery kit was placed at the basal interventricular septum 1 to 1.5 cm toward the apex, where pacing showed a positive QRS complex in lead DII, negative in DIII, and a “W” pattern in lead V1. At that site, perforation of the septum was performed so that the lead would reach the left bundle branch area.

Successful HB capture was defined as a QRS narrowing of more than 25% and a change in morphology from non-selective to selective HBP or non-selective HBP to myocardial capture with decremental pacing amplitudes (Figure 1c).

LBBAP was confirmed when the paced QRS complex had a qR or an RBBB morphology in lead V1 (Figure 2b) and one or more of the following criteria:

- Transition from non-selective to selective or myocardial capture with decremental pacing amplitudes.
- Presence of a left bundle branch/fascicular potential.
- Pacing stimulus to R wave peak time in lead V6 duration < 80 ms.
- Extrastimuli pacing to prove the capture of multiple structures by unmasking their different refractory periods (Figure 2b).

No backup ventricular pacing leads were used for both HBP and LBBAP strategies.

All the procedural characteristics were recorded.

### 2.3. Follow-Up

The follow-up visits after the procedure were performed in the outpatient clinic at 1, 3, and 6 months and then every 6 months. Sensing and pacing thresholds, as well as late complications, were recorded during follow-up.

### 2.4. Statistical Analysis

Continuous data were presented as mean ± one standard deviation, and categorical data were presented as frequencies and percentages. The normality of data distribution was assessed across all study groups. The t-test for independent or dependent groups was used to compare means for samples with normally distributed population data. Otherwise, the Mann–Whitney U-test for independent groups or the Wilcoxon test for dependent groups were used. The relationship between two categorical variables was evaluated with Fischer’s exact test. The association between continuous and dichotomous variables was assessed using binomial logistic regression. A 95% confidence interval was considered for all tests, and a *p* < 0.05 was interpreted as statistically significant. Statistical analysis was performed using SPSS software v 26.0 (IBM, Armonk, NY, USA).

### 2.5. Ethical Aspects

The study received approval from the institutional ethics committee and was conducted according to the recommendations of the 2013 revised Declaration of Helsinki. Before the cardiac pacing procedure, all patients were informed and provided written consent.

## 3. Results

### 3.1. Baseline Characteristics

The baseline characteristics of the entire study group, with a comparison between the HBP and the LBBAP patients, are shown in Table 1. A total of 68.3% of the patients had an LBBB morphology. The main indications for cardiac pacing for both groups were atrioventricular block and resynchronization therapy. Left and right atrial volumes and RV diameter values were significantly higher in the LBBAP arm. Both groups had similar comorbidities and medical treatment.

By comparison with RBBB, patients with LBBB had a significantly higher right atrial volume (47.30 ± 23.10 mL vs. 43.95 ± 14.50 mL, *p* = 0.04), a higher end-diastolic LV volume (158.41 ± 82.31 mL vs. 113.64 ± 33.55 mL, *p* = 0.03), and a lower ejection fraction (34.61 ± 13.90% vs. 53.14 ± 9.85%, *p* = 0.005).

### 3.2. Procedural Characteristics

A comparison of procedural characteristics between HBP and LBBAP is presented in Table 2. The global procedural success for CSP was 93%. In the end, 51 patients received HBP, and 43 patients received LBBAP. HBP had a significantly lower success rate than LBBAP (50.5% vs. 86%). The main reasons for procedural failure with HBP were the inability to identify the HB electrogram, HB capture failure, or capture at unacceptably high thresholds, while LBBAP failure was due to the inability to penetrate the interventricular septum. The distribution of BBB morphologies was similar between the two groups, but the native QRS duration was wider in the LBBAP group. The paced QRS duration was narrower with HBP, and more patients in this group had a QRS duration smaller than 130 ms. The pacing and sensing thresholds were significantly better with LBBAP, and there was no difference in fluoroscopy time or procedural duration. Procedural complications occurred only in the LBBAP group (two acute perforations in the LV cavity and one acute chest pain during lead fixation) without long-term sequelae. One patient required reintervention for lead repositioning before discharge.

The procedural characteristics between the two types of BBB are depicted in Table 3. The procedural success rate was higher in the RBBB versus LBBB group without reaching statistical significance for both HBP (62.5% vs. 44.9%) and LBAP (100% vs. 81.5%). The rest of the parameters showed no significant differences.

As HBP was the first pacing option, we assessed its procedural outcome relationship with several clinical, electrocardiography, and echography parameters. Binary logistic regression identified the baseline QRS duration, atrial volumes, and RV diameters as the only factors significantly associated with HBP procedural failure (Table 4).

### 3.3. Follow-Up

The follow-up period was longer for HBP than for LBBAP. LBBAP maintained significantly better pacing and sensing thresholds at the end of the follow-up period. Seven patients in the HBP group had an increase in pacing thresholds of more than 1 V, compared to none in the LBBAP group. The follow-up pacing and sensing thresholds were similar for both BBB morphologies, and significant threshold rises were distributed equally among the two groups (Table 5).

**Figure 3:**
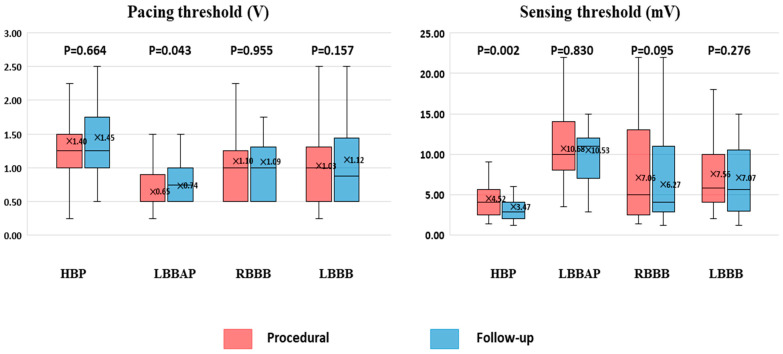
Comparison between the baseline and the follow-up values for all study groups.

A comparison between the baseline and the follow-up values for all study groups, according to the type of procedure and BBB morphology, is shown in Figure 3. There were no statistically significant differences except for a slight increase in LBBA pacing threshold and a decrease in ventricular sensing in patients with HBP.

From a clinical point of view, in patients without an indication of cardiac resynchronization therapy, the LV ejection fraction was preserved at the end of the follow-up period both in the HBP (54.37 ± 6.98% vs. 53.94 ± 6.39%, *p* = 0.693) group and the LBBAP group (49.49 ± 8.51% vs. 53.25 ± 8.32%, *p* = 0.006).

On the other hand, in patients with a baseline reduced ejection fraction (below 35%) and, therefore, with an indication of cardiac resynchronization therapy, the ejection fraction significantly increased in both pacing groups (from 25.93 ± 7.49% to 43.65 ± 7.34%, *p* < 0.001, in the HBP group, and from 23.92 ± 5.23% to 39.32 ± 9.04%, *p* < 0.001, in the LBBAP group).

Consequently, this impact on LV function translated into significantly better functional status, with an overall decrease in the NYHA class from 2.08 ± 0.82 to 1.53 ± 0.58 in the HBP group and from 2.14 ± 0.89 to 1.51 ± 0.55 (*p* < 0.001) in the LBBAP group.

### 3.4. Complications

Five patients (9.8%) had post-discharge complications in the HBP group. Three had premature battery depletion due to high pacing thresholds, one patient had an infection requiring device removal, and one had cardiac tamponade drained percutaneously without recurrence. One patient in the LBBAP group presented lead dislodgement, in which the repositioning of the lead was needed, and two patients had an infection with uncomplicated system removal.

## 4. Discussion

The main findings of this study were (I) in patients with baseline BBB, the success rate was significantly higher with LBBAP compared to HBP; (II) BBB correction was more significant with HBP; (III) there were no significant differences in procedural outcomes between LBBB and RBBB; (IV) left and right atrial volumes and RV diameter impacted the final result of HBP; (V) for successful CSP procedures, the pacing and sensing thresholds were stable over a medium-term follow-up period; and (VI) the complication rate that led to pacing interruption was low in all study groups.

### 4.1. Procedural Outcomes

The overall success rate for HBP in our study was 50.5%, significantly less than the values of around 80% reported in the general population [10]. Without statistical significance, HBP was less successful in LBBB than RBBB (44.9 vs. 62.5%). A similar finding was reported in the first randomized study with LBBB patients, where the success rate for correction was 52% [11]. The presence of a BBB is a marker of a diseased His–Purkinje system, and the ability to correct conduction abnormality is related to the proximity of conduction delay. Upadhyay et al. mapped the left-sided conduction system in patients with LBBB and proved that in only 64% of the patients, the site of the block was within the bundle of His or the proximal left bundle branch and, thus, amenable for correction [12]. Similar to our study, the success rate presented in previous studies with RBBB patients was higher for HBP, reaching a 78% correction rate [13].

On the other hand, the target in LBBAP is more distal and broader than that in HBP so that more patients can benefit. Our success rates of 81.5% in LBBB patients and 100% in RBBB patients are in line with the results of trials previously published in the literature. Wang et al. conducted a randomized efficacy trial between LBBAP and biventricular pacing for patients with an indication for resynchronization therapy. The reported success rate in the LBBAP arm was 90% [14]. Also, in a multicenter registry study, the success of LBBAP in patients with RBBB was 88% [15]. Finally, it is important to mention, in our study, that the superior success rate in RBBB for both procedures could have been influenced by the more structurally affected hearts in patients with LBBB, with larger right atria and left ventricles and a lower ejection fraction.

Correction with HBP generated a much narrower QRS complex compared to LBBAP. A meta-analysis also highlighted this by comparing QRS duration in different pacing modalities and proving that HBP was associated with the narrowest-paced QRS complex [16]. This is possible in patients with intra-His conduction delays, in which the capture of the distal fibers beyond the lesion site will lead to synchronous and fast ventricular depolarization through both bundle branches. In LBBAP, although the LV is activated physiologically through the left bundle branch, there is a delay in RV activation, which is responsible for widening the distal part of the QRS complex. If in LBBB, the argument for QRS narrowing is evident due to the capture of the dormant left bundle fibers, in RBBB, the mechanisms are less clear. The most plausible explanations at this moment are linked to the faster activation of the RV than in the native state, which occurs through one or both of two mechanisms: retrograde conduction through the left bundle, HB, and anterograde recruitment of the dormant right bundle branch fibers and direct septal capture and transseptal activation of the right side of the septum from the pacing site [17].

Both the fluoroscopy and the procedural times were non-significantly longer in the LBBAP arm since these patients underwent an initial attempt at HBP.

Our study adds to the constant findings across HBP studies that pacing thresholds are higher and sensing values are lower than those encountered in LBBAP [18]. This is related to the anatomy of the His bundle, which is encapsulated in a fibrous sheath and sometimes more challenging to capture. In LBBAP, the pacing threshold is usually similar to conventional right ventricular pacing, and because the lead is deep into the interventricular septum, the sensing values are excellent.

In a recently published MELOS study, the largest multi-center registry on LBBAP, the most common acute complication was perforation into the LV cavity in 3.7% of the patients [7]. In our study, this occurred in two patients (4.6%). Fortunately, this situation is benign, requiring lead withdrawal into the RV and perforation of the septum at another site. Acute chest pain is another complication reported in our study, without significant myocardial damage. Finally, lead dislodgement before discharge was encountered in one patient (2.3%), compared to 1.5% reported in the Melos registry [7].

We have shown in previous studies that both left and right atrial enlargements were significantly associated with failure to identify the HB electrogram when using a non-deflectable catheter and that baseline QRS duration was a prognostic factor for HBP procedural failure [19]. In the present study, although we used non-deflectable and deflectable catheters, atrial volumes, right ventricular diameter, and baseline QRS width were significantly associated with HBP procedural failure. Enlarged heart chambers, especially the right ones, modify the trajectory of the bundle, displacing it anteriorly and more apically, thus making it difficult to find and capture [20]. On the other hand, a larger QRS complex is an indirect marker of extensive myocardial dysfunction and probably of diffuse conduction system disease, making capturing the conduction system with HBP less likely. By comparison, LBBAP, although more difficult in dilated hearts, requires less precision than HBP. Even in significantly altered anatomies, the current delivery tools can easily achieve a perpendicular position on the mid-septum. Once the septum is perforated, the larger target area of left bundle ramifications offers more possibilities to engage the conduction system than HBP.

### 4.2. Follow-Up

The follow-up period for HBP was longer simply because we started conducting LBBAP approximately one year after beginning HBP. One of the concerns regarding permanent HBP in patients with an already abnormal conduction system is the possibility of disease progression distal to the pacing site, making the procedure redundant. Also, a significant limitation of the procedure is the risk of HB bundle capture threshold rise over time. Previous studies have reported an increase in the pacing threshold to higher than 2.5 V in up to 27% of the patients and loss of His bundle capture in 7.6% of the patients [21]. In our study, both pacing and sensing thresholds were stable over time. Only seven patients (13.7%) in the HPB group had a threshold rise of more than 1V without interruption of HBP, leading to premature battery depletion in three patients. We believe that the lower incidence of threshold rise in our study can be explained by the initial excellent pacing thresholds, which suggests adequate fixation of the lead in the conduction system, an important endpoint since we did not use any ventricular backup leads.

Similar to what has been described in previous studies that focused on patients with BBB and reduced ejection fraction, we also found that after successful CSP procedures (irrespective of the structure captured) and significant QRS narrowing, there is a significant increase in LV ejection fraction and, consequently, in functional status [11,13,14].

There was one lead dislodgment recorded in the LBBAP group. None of the patients in the LBBAP group had significant changes in ventricular thresholds, and the overall values remained better in this group, offering arguments that LBBAP is a more attractive option than HPB.

The type of BBB morphology did not influence the mid-term evolution of pacing and sensing parameters, both of which remained constant over time. Furthermore, there was an equal distribution of complications in the two groups.

To our knowledge, this is the only comparative study for CSP outcomes between left and right bundle branch block patterns after a medium-term follow-up period.

### 4.3. Study Limitations

Some limitations of the study should be mentioned. The retrospective collection of data limits the strength of the evidence. The first part of the cases was performed during our learning curve. Although it may not impact the overall comparison of the data between different groups, the values of procedural parameters may improve after significant experience. Also, the study addressed patients with typical BBB. We excluded patients with non-specific intraventricular conduction delays since these patients may respond less to CSP. For the patients included in the study, we performed LBBAP as a bailout strategy for HBP. Had we used LBBAP as the first strategy, the success rates may have differed. Also, in a larger cohort, the differences in success rates between LBBB and RBBB patients could become significant. The follow-up period for LBBAP was shorter than for HBP, so later complications could have been missed in these patients.

The key point of this study evaluating LBBB vs. RBBB patients is that once CSP capture is achieved with low pacing thresholds, the risk of future complications leading to CSP interruption is low, at least in the medium term. This also alleviates the need for a backup ventricular pacing lead, increasing procedural costs and risk. Given the frequently associated cardiac structural modifications in this population, other forms of pacing may accelerate the decline in LV function, making physiological pacing an attractive and feasible option in these patients.

## 5. Conclusions

In BBB patients, CSP is a feasible procedure associated with a high success rate, stable pacing and sensing parameters, and low complication rates over a mid-term follow-up.

## Figures and Tables

**Table 1:** Baseline patient characteristics.

BASELINE CHARACTERISTICS	All	HBP	LBBAP	*p*-Value
Age (years, mean ± SD)	68.31 ± 10.95	70.14 ± 8.68	66.14 ± 12.92	0.089
Male (N, %)	63 (62.3)	32 (62.7)	26 (60.5)	0.835
BMI (Kg/m^2^, mean ± SD)	28.36 ± 4.76	28.42 ± 5.4	28.28 ± 3.9	0.890
QRS duration (ms, mean ± SD)	151.7 ± 14.5	148.29 ± 14.33	155.74 ± 13.77	0.012
LBBB (N, %)	69 (68.3)	31 (60.8)	31 (72.1)	0.280
eGFR (mL/min, mean ± SD)	61.38 ± 19.73	60.93 ± 18.35	61.9 ± 21.46	0.814
NYHA class	2.11 ± 0.85	2.08 ± 0.82	2.14 ± 0.89	0.732
PACING INDICATION				
AV block (N, %)	45 (44.5)	23 (45.1)	21 (48.8)	0.836
Slow conducting AF (N, %)	7 (6.9)	2 (3.9)	3 (7)	0.657
Sick sinus syndrome (N, %)	8 (7.9)	6 (11.8)	1 (2.3)	0.120
Resynchronization therapy (N, %)	41 (40.6)	20 (39.2)	18 (41.9)	0.835
ECHOCARDIOGRAPHY				
LA volume (mL, mean ± SD)	68.46 ± 34.51	53.74 ± 14.29	82.8 ± 41.9	<0.001
RA volume (mL, mean ± SD)	46.18 ± 20.55	39.27 ± 11.84	53.1 ± 24.85	0.003
LVEDV (mL, mean ± SD)	145.2 ± 74.06	136.52 ± 46.82	152.1 ± 90.2	0.419
LVESV (mL, mean ± SD)	88.8 ± 62.53	81.42 ± 42.52	94.71 ± 74.86	0.414
EF (%, mean ± SD)	40.92 ± 15.39	43.2 ± 15.7	38.19 ± 14.7	0.115
RV diameter (mm, mean ± SD)	31.52 ± 4.85	30.11 ± 4.68	33.15 ± 4.58	0.004
Mitral regurgitation (grade, mean ± SD)	1.93 ± 1.09	1.94 ± 0.92	1.91 ± 1.27	0.884
Tricuspid regurgitation (grade, mean ± SD)	1.48 ± 0.98	1.5 ± 0.82	1.47 ± 1.14	0.869
COMORBIDITIES				
Hypertension (N, %)	85 (84.16)	49 (96.1)	33 (76.7)	0.01
Diabetes mellitus (N, %)	27 (26.73)	14 (27.5)	10 (23.3)	0.812
Ischemic disease (N, %)	36 (35.64)	16 (31.4)	17 (39.5)	0.515
Paroxysmal AF (N, %)	20 (19.8)	10 (19.6)	10 (23.6)	0.801
Permanent AF (N, %)	10 (9.9)	3 (5.9)	4 (9.3)	0.698
TREATMENT				
RAAS antagonists (N, %)	70 (69.3)	33 (64.7)	35 (81.4)	0.104
Beta-blockers (N, %)	78 (77.2)	41 (80.4)	37 (86)	0.585
MRAs (N, %)	45 (44.5)	20 (39.2)	25 (58.1)	0.097
SGLT-2 inhibitors (N, %)	9 (8.9)	1 (2)	4 (9.3)	0.175
Anticoagulants (N, %)	35 (34.6)	15 (29.4)	17 (39.5)	0.383

HBP—His bundle pacing; LBBAP—left bundle branch area pacing; SD—standard deviation; BMI—body mass index; LBBB—left bundle branch block; eGFR—estimated glomerular filtration rate; NYHA Class—New York Heart Association functional class; AV—atrioventricular; AF—atrial fibrillation; LA—left atrium; RA—right atrium; LVEDV—left ventricular end-diastolic volume; LVESV—left ventricular end-systolic volume; EF—ejection fraction; RV—right ventricle; RAAS—renin–angiotensin–aldosterone system; MRA—mineralocorticoid receptor antagonist; SGLT-2—sodium-glucose cotransporter-2.

**Table 2:** Procedural characteristics according to the pacing method.

	HBP	LBBAP	*p*-Value
Number of patients	51	43	
Procedural success (%)	50.5%	86%	<0.001
Baseline QRS duration (ms)	148.29 ± 14.33	155.74 ± 13.77	0.012
LBBB (N, %)	31 (60.8)	31 (72.1)	0.280
Paced QRS duration (ms)	100.49 ± 21.54	124.98 ± 15.80	<0.001
QRS > 130 ms (N, %)	4 (7.84)	12 (27.9)	0.013
Fluoroscopy time (min)	10.39 ± 9.85	13.72 ± 9.90	0.108
Procedural duration (min)	142.40 ± 41.13	152.46 ± 48.30	0.286
IMPLANT CHARACTERISTICS			
CSP capture threshold (V)	1.39 ± 0.75	0.65 ± 0.26	<0.001
R wave detection (mV)	4.52 ± 2.86	10.67 ± 5.10	<0.001
Pacing impedance (Ohm)	463.49 ± 97.29	546.65 ± 154.18	0.003
Periprocedural complications (N, %)	0 (0)	3 (7) ^a^	0.092
Lead revision before discharge (N, %)	0 (0)	1 (2.3) ^b^	0.457

^a^ Two acute perforations in the LV cavity and one acute chest pain during lead fixation; ^b^ one lead dislodgement. HBP—His bundle pacing; LBBAP—left bundle branch area pacing; LBBB—left bundle branch block; CSP—conduction system pacing; LV—left ventricle.

**Table 3:** Procedural characteristics according to the type of bundle branch block.

	LBBB	RBBB	*p*-Value
Number of patients	69	32	
HBP procedural success (%)	44.9%	62.5%	0.134
LBBAP procedural success (%)	81.5%	100%	0.174
Baseline QRS duration (ms)	158.29 ± 10.87	138.94 ± 11.92	<0.001
Paced QRS duration (ms)	113.34 ± 20.97	108.13 ± 25.58	0.294
Paced QRS > 130 ms	10 (16.1)	6 (18.7)	0.777
Procedural duration (min)	149.50 ± 43.76	144.42 ± 48.40	0.612
Fluoroscopy time (min)	11.11 ± 8.41	11.54 ± 12.58	0.818
IMPLANT CHARACTERISTICS			
CSP capture threshold (V)	1.03 ± 0.68	1.10 ± 0.70	0.631
R wave detection (mV)	7.56 ± 4.88	7.06 ± 5.55	0.660
Pacing impedance (Ohm)	488.05 ± 139.36	527.66 ± 115.72	0.171

LBBB—left bundle branch block; RBBB—right bundle branch block; HBP—His bundle pacing; LBBAP—left bundle branch area pacing; CSP—conduction system pacing.

**Table 4:** Binary logistic regression for HBP procedural failure.

Parameters	OR	95% CI	*p*-Value
Age (years)	0.966	0.929–1.004	0.082
Sex	1.101	0.478–2.536	0.821
BMI (kg/m^2^)	0.994	0.912–1.083	0.888
eGFR (mL/min)	1.003	0.982–1.023	0.812
Type of BBB	0.600	0.251–1.435	0.251
Baseline QRS duration (ms)	1.039	1.007–1.072	0.015
Ejection fraction (%)	0.979	0.952–1.005	0.116
LVEDV (mL)	1.003	0.996–1.011	0.419
LVESV (mL)	1.004	0.995–1.013	0.414
LA volume (mL)	1.038	1.014–1.063	0.002
RA volume (mL)	1.041	1.011–1.071	0.007
RV diameter (mm)	1.157	1.042–1.285	0.006
Mitral regurgitation	0.971	0.668–1.412	0.879
Tricuspid regurgitation	0.964	0.632–1.470	0.865
AF	1.411	0.576–3.458	0.451

OR—odds ratio; CI—confidence interval; BMI—body mass index; eGFR—estimated glomerular filtration rate; BBB—bundle branch block; LVEDV—left ventricular end-diastolic volume; LVESV—left ventricular end-systolic volume; LA—left atrium; RA—right atrium; RV—right ventricle; AF—atrial fibrillation.

**Table 5:** Follow-up parameters.

	**HBP**	**LBBAP**	***p*-Value**
Follow-up duration (days)	790.43 ± 446.55	595 ± 346.42	0.028
CSP capture threshold	1.44 ± 0.86	0.73 ± 0.24	<0.001
R wave detection	3.47 ± 1.97	10.52 ± 4.40	<0.001
Threshold increase > 1 V	7 (13.72)	0 (0)	0.014
Reinterventions for lead revision	3 (5.88) ^a^	1 (2.32) ^b^	0.622
Other complications	2(3.92) ^c^	2 (4.65) ^d^	1
	**LBBB**	**RBBB**	***p*-Value**
Follow-up duration (days)	686.67 ± 432.61	739.29 ± 383.68	0.589
CSP capture threshold	1.11 ± 0.76	1.09 ± 0.67	0.880
R wave detection	7.07 ± 4.73	6.27 ± 5.16	0.467
Threshold increase > 1 V	3 (4.8)	4 (12.5)	0.417
Other complications	2 (3.2) ^e^	2 (6.2) ^f^	1

^a^ Box change for premature battery depletion; ^b^ one lead dislodgement; ^c^ one device infection and one cardiac tamponade; ^d^ two device infections; ^e^ one device infection and one cardiac tamponade; ^f^ two device infections. HBP—His bundle pacing; LBBAP—left bundle branch area pacing; LBBB—left bundle branch block; RBBB—right bundle branch block; CSP—conduction system pacing.

## Data Availability

The datasets are available upon reasonable request to the corresponding author.
